# Interindividual variability in static apnoea performance is partly explained by genetic factors

**DOI:** 10.1113/EP093374

**Published:** 2026-06-19

**Authors:** Anastasios Makris, Alexandros Sotiridis, Nickos Geladas, Kalliopi Gkouskou, Vassilis Klissouras, Maria D. Koskolou

**Affiliations:** ^1^ Section of Sports Medicine and Biology of Exercise, School of Physical Education and Sport Science National and Kapodistrian University of Athens Athens Greece; ^2^ Department of Biology, School of Medicine National and Kapodistrian University of Athens Athens Greece

**Keywords:** diving response, face immersion, heritability, interindividual variability, static apnoea, twin model

## Abstract

A large interindividual variability exists in maximal apnoea duration and the physiological adjustments that occur during apnoea. Although a few genes have been found to be associated with some of the cardiovascular responses during apnoea, the extent to which genetic and environmental factors contribute to individual differences in apnoea performance has not been studied. Accordingly, 12 monozygotic and 10 dizygotic twin pairs (*n* = 44) performed a series of five maximal apnoeas with the face immersed in cold water to determine the heritability estimates for apnoea duration and the accompanying haemodynamic adjustments. Heritability estimates were derived and showed that genetic factors explain a substantial portion of the interindividual differences in maximal apnoea duration (82%) and in the mean arterial pressure (74%) and diastolic pressure (73%) responses to apnoea. Additionally, genetic influences accounted for 77% of the variability in forced vital capacity. Nonetheless, the heritability estimates for heart rate, systolic pressure, stroke volume, cardiac output and total peripheral resistance could not be calculated, since the statistical assumptions required to estimate genetic variance were not met. Overall, it appears that a large portion of the variability observed in maximal static apnoea duration and the arterial pressure response can be explained by genetic differences. It should be emphasised that heritability estimates reflect the proportion of the variance for a trait and do not imply biological determinism. The significant heritability observed for apnoea duration likely reflects contributions from multiple genetically influenced physiological systems but might also be influenced by unmeasured shared environmental factors.

## Abstract (highlights)

- **What is the central question of this study?**
  Maximal apnoea duration and the accompanying physiological adjustments show significant interindividual variation. However, the extent to which genetic differences explain this variation has not been previously explored.
- **What is the main finding and its importance?**
  Using the twin model, we show that a substantial portion of the interindividual variation in apnoea duration and the arterial pressure responses to apnoea is explained by genetic factors.

## 1 INTRODUCTION

Prolonged episodes of voluntary breath‐holding (apnoea) occur in various sporting (e.g., underwater rugby, artistic swimming), recreational (e.g., spearfishing) and occupational [e.g., sea‐food freedivers, such as the Japanese (Ama) and Korean (Haenyeo) divers] activities. Maximal breath‐hold duration depends on the magnitude of apnoea‐induced physiological responses (i.e., the magnitude of cardiovascular adjustments) and on psychological factors (Hoiland et al., 2017; Schneider, 1930) and shows a large interindividual variability (Godfrey & Campbell, 1968; Parkes, 2006). However, it is not known to what extent genetic factors contribute to this variability.

The primary physiological adjustments that occur during apnoea, collectively called the cardiovascular diving response, include vagally mediated bradycardia and sympathetically mediated peripheral vasoconstriction (Heistad et al., 1968; Leuenberger et al., 2001; Perini et al., 2008; Schagatay & Andersson, 1998). An augmented response is developed when simultaneously immersing the face in cold water (Fagius & Sundolf, 1986; Schagatay & Holm, 1996), which elicits a diving reflex (Lemaitre et al., 2015). The diving reflex refers specifically to the trigeminocardiac reflex elicited by facial immersion without obligatory breath‐holding, whereas the diving response represents a broader integrative physiological phenomenon involving voluntary breath‐holding, chemoreflex stimulation, autonomic cardiovascular adjustments and mechanical effects of changes in lung volume (Elia & Gennser et al., 2021). From a teleological perspective, the diving response acts as an oxygen‐conserving mechanism, as bradycardia reduces myocardial oxygen consumption, and vasoconstriction of the peripheral beds centralises oxygen‐rich blood flow to the most vital organs (Andersson et al., 2004; Heistad et al., 1968; Lindholm et al., 1999). The progressively increasing vasoconstriction is proportionately greater than the chronotropic decrease in cardiac output and, consequently, progressive hypertension develops (Andersson et al., 2000). Although this hypertensive response is typically observed during dry apnoeas or during apnoeas with only the face immersed, limited evidence in trained divers suggests that it may be attenuated during apnoea with whole‐body submersion (Sieber et al., 2009). Similar to maximal apnoea duration, large interindividual differences in the magnitude of these responses also exist. Apnoea training has been shown to influence these responses, with training studies reporting enhanced diving bradycardia and peripheral vasoconstriction during apnoea following a period of apnoea training (Engan et al., 2013; Schagatay et al., 2000) and with cross‐sectional studies indicating that trained divers exhibit a stronger diving bradycardia (Bakovic et al., 2003; Elia & Barlow et al., 2021) and higher arterial blood pressure at the end of apnoea (Heusser et al., 2009) compared with non‐divers. Beyond training status, variability in apnoeic responses may also arise from differences in inspiratory lung volume at apnoea onset (Song et al., 1969), pre‐apnoeic breathing protocols and associated hypocapnia (Hill, 1973), dietary composition and recent nutritional intake (Elia et al., 2022), and menstrual cycle phase in female participants (Cherouveim et al., 2020).

Accumulating evidence suggests that part of this variability in apnoeic responses could also be explained by genetic variation (Aguilar‐Gómez et al., 2025; Baranova et al., 2017). Baranova et al. (2017) have thus observed that individuals with polymorphisms in the bradykinin receptor B2 (*BDKRB2*) and the angiotensin‐converting enzyme (*ACE*) genes, two genes that express proteins related to vasomotor activity, exhibited the highest blood pressure changes during maximal static apnoea. Interestingly, a single nucleotide polymorphism (SNP) located just upstream from *BDKRB2* has also been shown to be prevalent in the Bajau of Indonesia, a population of indigenous freedivers (Ilardo et al., 2018).

Maximal voluntary apnoea is a polygenic trait as it recruits complex and interacting physiological mechanisms and, most probably, many more relevant gene polymorphisms exist. Without presuming which genes are involved, a twin‐study design allows us to unravel the relative contribution of environmental and genetic factors in the variation observed in any phenotype (Bouchard et al., 2011; Klissouras, 1971). Based on the twin model and the comparison of intrapair differences between monozygotic (MZ) and dizygotic (DZ) twins, heritability estimates (*h* ^2^) can be derived, which signify the amount of phenotypic variance ascribed to genetic differences (Klissouras et al., 2007). Together with genome‐wide association studies, determination of heritability estimates could provide an insight into the variation observed in the performance of exceptional athletes and individuals who manifest divergent apnoea‐related physiological features.

To date, no study has reported heritability estimates with respect to maximal static apnoea duration and the accompanying cardiovascular adjustments. Therefore, the purpose of the present study was to assess the relative contribution of genetic and environmental factors to the variation observed in maximal static apnoea duration and the cardiovascular diving response by comparing intrapair differences between MZ and DZ twins.

## 2 MATERIALS AND METHODS

### 2.1 Ethics approval

This study was approved by the Research Ethics Committee of the Department of Physical Education and Sport Science of the National and Kapodistrian University of Athens, Greece (1452/11‐01‐2023), and all experimental procedures conformed to the *Declaration of Helsinki*, except for registration in a database. All participants received a detailed description of the experimental procedures of the study and provided their written informed consent before their participation.

### 2.2 Participants

A total of 44 male twins (12 MZ and 10 DZ pairs), 18–33 years old (24.7 ± 5.3 and 24.7 ± 4.4 years, respectively; mean ± SD) participated in this study. All but one (from an MZ twin pair; type I diabetes) of the participants were not suffering from any cardiovascular, neurological, respiratory or metabolic diseases. Nine of the participants were regular smokers (in one MZ and one DZ twin pair, both siblings were smokers, whereas in one MZ and four DZ pairs, only one sibling was a smoker).

### 2.3 Equal‐environmental assumption

Environmental comparability is an essential assumption made in the twin model; this assumption, however, cannot be tested. Efforts were therefore made in the present study to partially address potential environmental confounding by assessing a range of lifestyle and activity‐related factors. Participants completed a detailed questionnaire regarding fitness level, physical activity during work hours, sports participation and some other leisure‐time physical activities, with five options ranging from ‘almost never’ to ‘very much’ (Baecke et al., 1982).

Environmental contributions to phenotypic variance can be divided into shared environmental factors, which are common to both twins, and non‐shared environmental factors, which are unique to each sibling. Residual environmental variance arising from unaccounted environmental influences might bias heritability (*h* ^2^) estimates by altering within‐pair variance in MZ and DZ twins.

If residual environmental differences have a meaningful effect on apnoea performance and are large within MZ twin pairs, this would increase within‐pair variance among MZ twins and lead to a downward bias in *h* ^2^ estimates. In contrast, an upward bias in *h* ^2^ might occur if MZ twins experience more similar environmental exposures than DZ twins; for example, through more similar parental or social treatment. If such environmental similarity affects apnoea performance, it would reduce within‐pair variance in MZ twins relative to DZ twins, thereby inflating heritability estimates.

The opposite pattern applies to DZ twins. If residual environmental differences that might affect apnoea performance are large primarily within DZ twin pairs, this would increase within‐pair variance among DZ twins relative to MZ twins, resulting in an upward bias in *h* ^2^ estimates.

However, twins are generally exposed to highly similar environments, particularly during early adulthood, with environmental divergence increasing with age. Given that the participants in the present study were relatively young men (mean age 24 years), large residual environmental differences between twin siblings are unlikely. Although residual environmental effects cannot be excluded, their influence on the estimation of heritability for apnoea performance in this study is expected to be limited. Moreover, a question regarding their experience in diving activities was added for the specific purpose of the present study. Twenty pairs (11 MZ and 9 DZ pairs) had no experience in diving‐related activities, whereas two pairs (one MZ and one DZ pair) were matched for diving experience based on the years and types of diving‐related activities (spearfishing and free‐diving, respectively).

### 2.4 Zygosity

A questionnaire was administered to determine the zygosity of the twin pairs (i.e., to classify them as identical or non‐identical twins). The questionnaire was divided into two parts. The first part, completed by the investigators observing details in the appearance of twins, included questions related to the similarity in relevant morphological and physical characteristics, including eye colour, hair colour, hair texture, facial appearance, shape of ear lobes, the ability to roll the tongue, weight and height. The second part included two questions related to their resemblance during childhood: (1) whether they would be described as (a) ‘alike as two peas in a pod’, (b) as ‘alike as ordinary siblings’, or (c) as ‘not alike at all’; and (2) whether at school age, people at school, parents, close friends or strangers had difficulty telling them apart. For this part of the questionnaire, we asked the twin brothers to answer the questions in separate rooms (in order to avoid interference between the two brothers). The same questionnaire has been used in previous studies from our laboratory (Missitzi et al., 2008, 2018). Both parts of the questionnaire have repeatedly been shown to determine zygosity with high accuracy, with reported accuracies of 92% for MZ and 97% for DZ twins (Chen et al., 1999; Fairpo, 1979; Jarrar et al., 2018; Kasriel & Eaves, 1976; Ooki et al., 1990).

Misclassification of twin zygosity typically leads to lower heritability estimates. If an MZ pair is misclassified as DZ, which is the most common form of misclassification, this would decrease within‐pair variance in the DZ group and bias heritability (*h* ^2^) estimates downwards. Likewise, if a DZ pair is misclassified as MZ, this would increase within‐pair variance within the MZ group and also bias heritability (*h* ^2^) estimates downwards.

It is common practice to classify twins as MZ or DZ based on the number of placentae at birth. However, there are many instances where this method might lead to misclassification. During the very early stages of pregnancy, an embryo carrying MZ twins may split in ∼25% of cases, which can later lead to misclassification when ultrasound examination is performed (Ooki et al., 2004).

In the present study, two twin pairs were raised with the understanding that they were DZ; however, responses to the questionnaire revealed no detectable differences between the twins, raising concerns regarding their zygosity. Thus, for these twin pairs, DNA analysis was performed. Each twin received two buccal swabs for saliva sample collection. To ensure adequate DNA collection, participants were instructed to rub the inside of their cheek firmly for a minimum of 1 min. This approach was chosen over shorter durations (e.g., 15 s) or a predetermined number of rubs to maximise DNA yield. Genomic DNA was extracted from the buccal swabs using the Invitrogen™ DNA Mini Kit (Invitrogen, Germany), following the manufacturer's protocol. Total DNA concentration was quantified using a Qubit™ fluorometer (Thermo Fisher Scientific) according to the manufacturer's instructions. The extracted DNA was stored at −20°C until further processing.

Samples were prepared for SNP genotyping using the Illumina Infinium HTS Assay, following the Infinium HTS Assay Reference Guide and manual protocol. SNP genotyping was performed using the Infinium Global Screening Array‐24 v.3 BeadChip (Illumina, CA, USA), which allows for the analysis of ≤750 000 SNPs and copy number variation markers per sample. All BeadChips were scanned using the Illumina iScan System array scanner according to the manufacturer's guidelines.

The results of the DNA analysis revealed that the two pairs of twins were genetically identical (MZ). Systematic genotyping of all twin pairs was not performed owing to financial constraints.

### 2.5 Experimental protocol

The participants visited the laboratory after refraining from caffeine‐ and alcohol‐containing beverages for >12 and >24 h, respectively. Within each pair of twins, the brothers were asked to follow similar diets for 12 h prior to their visit. In 18 twin pairs (9 MZ and 9 DZ), the brothers (twin A and twin B) visited the laboratory together, undertaking the experiment on the same day with ∼1 h difference. In four twin pairs (three MZ and one DZ), the brothers (twin A and twin B) visited the laboratory on different days <1 week apart, but at similar times of the day. In all cases, we required that the brothers remained unaware of each other's performance until the completion of the experimental procedure for both.

**FIGURE 1:**
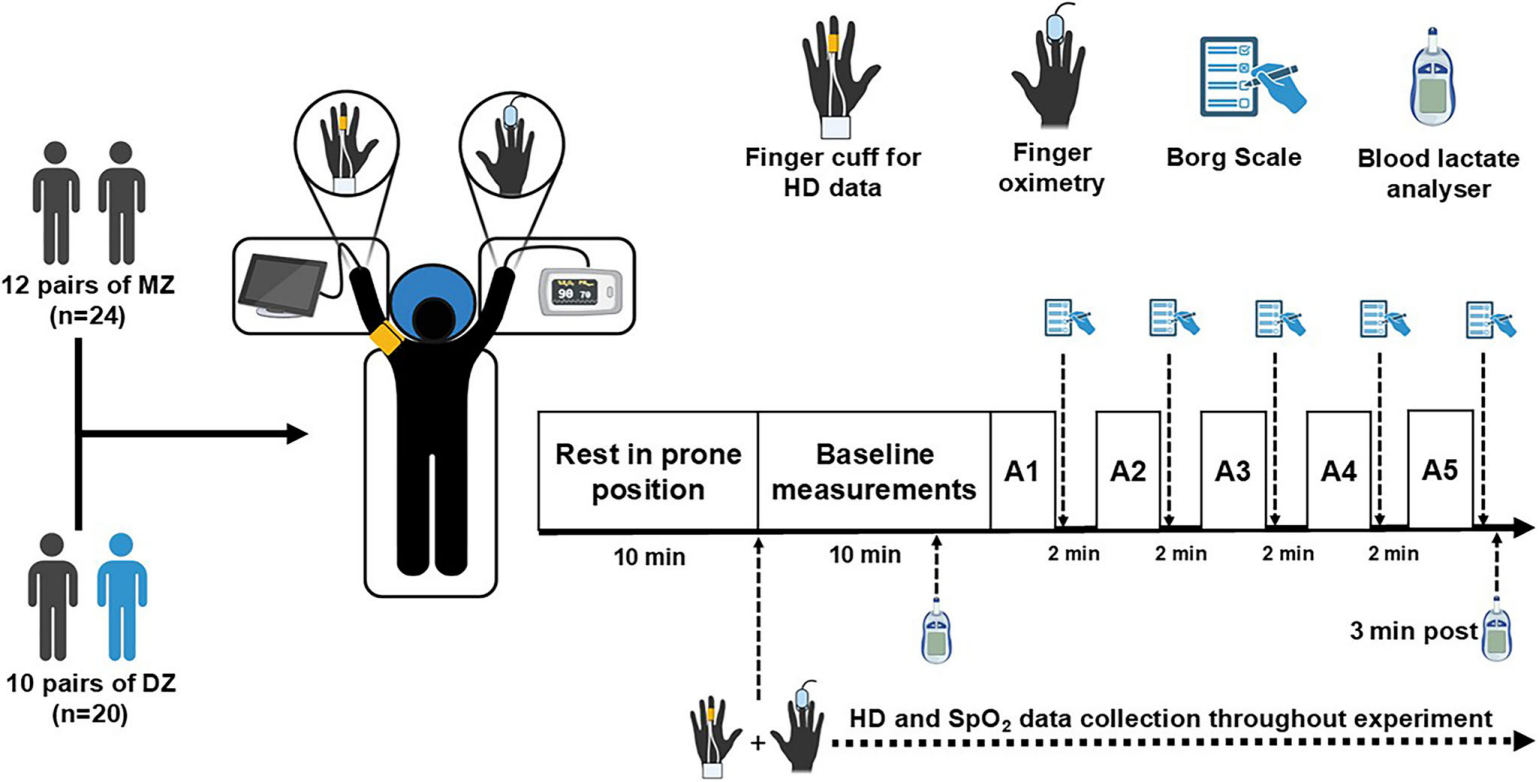
A schematic representation of the experimental protocol. Abbreviations: A, apnoeic trial; DZ, dizygotic; HD, haemodynamic; MZ, monozygotic; $S_{pO_{2}}$, peripheral oxyhaemoglobin saturation.

Upon arrival at the laboratory, the participant's anthropometric measurements were collected (height and body mass, Bilance salus, Milano) and the zygosity and physical activity questionnaires were administered. When completed, forced vital capacity (FVC) was measured in a sitting position using a metabolic cart (MedGraphics CPX/Ultima, Saint Paul, MN, USA) following the updated spirometry standards (Graham et al., 2019), with at least three acceptable manoeuvres performed, a difference of ≤0.150 L required between the two highest FVC values, and the largest FVC value obtained. The participant then rested for 10 min in a prone position on a mattress with the head resting on rigid cover, under which a water container was placed. The participant's arms were positioned on two mattress extensions on both sides of the water container (Figure 1). We included this resting period to establish stable cardiovascular variables. During the same period, the participant was given the instructions on how to perform the repeated apnoea protocol. Another 10 min period followed, wherein all instruments were applied on the participant, and baseline data were collected. A series of five maximal apnoeas followed, with the face immersed in cold water. To standardise lung volume before each apnoea, participants exhaled to residual volume, then inhaled a volume of air corresponding to 80% of their individually measured sitting FVC.

The participant was notified 60 s before the apnoeic bout and instructed to avoid hyperventilation during this period. A nose clip was attached 30 s before each apnoeic episode, and 10 s before, he raised his head, and the rigid cover was removed from the water container. Following a 5 s countdown, the participant exhaled to his residual volume and inhaled a volume of air equivalent to 80% of his sitting FVC through a mouthpiece that was connected to a rubber air bag, previously filled with room air using a calibrated syringe. The moment the rubber bag was completely emptied by a single inspiration, the mouthpiece was removed, marking the start of the apnoeic episode, with the participant flexing his head and immersing his entire face (chin and forehead) in cold water. He was instructed to volitionally hold his breath for as long as possible, to neither swallow nor exhale in the water and to avoid unnecessary movements during breath‐holding. The water and ambient air temperatures were 10°C (9°C–11°C) and 22°C (21°C–23°C), respectively. Termination of apnoea was defined as the moment the participant started exhaling after raising his head out of the water. A total of five repeated maximal apnoeas were performed, interspersed with 2 min intervals during which the rigid cover was placed back on the water container, the nose‐clip was removed, and the face was dried using paper towels.

To ensure that participant gave his best effort, a Borg scale (Borg, 1970) rating of 18–20 was required at the end of each apnoea.

### 2.6 Experimental measures

Measurements of cardiovascular parameters and peripheral O_2_ saturation began 5 min prior to the first apnoeic episode and continued until 2 min after the end of the experiment. Systolic pressure (SP), diastolic pressure (DP), mean arterial pressure (MAP), heart rate (HR), stroke volume (SV), cardiac output (*Q̇*) and total peripheral resistance (TRP) were recorded continuously using a photoplethysmometer, with the cuff on the left middle finger (Finometer Nova Plus, Finapres Medical Systems, Amsterdam, The Netherlands). Peripheral oxyhaemoglobin saturation ($S_{pO_{2}}$) was measured using a finger pulse oximeter (NELLCOR Symphony N3000, USA), with the probe attached on the right middle finger. At baseline and 3 min following the last apnoeic episode, a drop of finger‐capillary blood was sampled for the measurement of blood lactate concentration [La] using a lactate analyser (Lactate Scout 4, EKF diagnostics). Apnoea duration was measured in seconds with an electronic stopwatch.

### 2.7 Data analysis

Baseline values for SP, DP, MAP, HR, SV, *Q̇*, TPR and $S_{pO_{2}}$ were calculated as 3 min mean values from the first 3 min of the 5 min baseline period preceding the apnoeic series. Baseline blood [La] was also obtained during this period.

The repeated apnoea protocol was used to ensure that the participants achieved their best apnoeic performance. For each participant, only the apnoeic bout with the longest duration was chosen for the analysis of all cardiovascular variables. Apnoeic values for HR (end‐apnoeic HR) blood pressure (end‐apnoeic SP, end‐apnoeic DP and end‐apnoeic MAP), SV (end‐apnoeic SV), *Q̇* (end‐apnoeic *Q̇*) and TPR (end‐apnoeic TPR) were calculated as mean values of the period 20–10 s before the end of the apnoeic bout with the longest duration. HR and MAP were additionally analysed in all apnoeic episodes to elucidate trial‐by‐trial descriptives. It should be noted that no universal approach exists in the determination of diving bradycardia. A commonly used approach is to calculate the mean HR of the apnoeic episode, excluding values from the initial 30 s (Schagatay et al., 1999, 2007). In addition, the average values for the last 10 s of apnoea have been used as diving bradycardia (Andersson et al., 2004), whereas others have used the lowest HR value during apnoea (Lemaitre et al., 2008; Mulder et al., 2025). Given that the cardiovascular diving response is dynamic, with some cardiovascular parameters changing in opposite directions and in different phases within apnoea (Perini et al., 2008), we used the mean values of the period 20–10 s before the end of the apnoeic bout because cardiovascular adjustments appear relatively more stable during this period (Persson et al., 2023). In addition, both psychological and physiological stress are likely to be more pronounced during this late phase of apnoea.

The nadir $S_{pO_{2}}$ value observed during the first 30 s after the end of the longest apnoeic bout was used as the apnoeic $S_{pO_{2}}$; this nadir reflects the end of apnoea, as there is a circulation delay of ∼20–30 s, when finger pulse oximetry is used (Severinghaus & Naifeh, 1987).

### 2.8 Statistical analysis and heritability estimates

Data are reported as the mean ± SD. Apnoea duration, haemodynamic and lactate data were analysed across all participants collectively (*n* = 44), irrespective of twin type, to assess the effect of apnoea repetition on apnoea duration and the effect of apnoea on haemodynamic and lactate responses. Single‐factor ANOVA was applied to determine the effect of apnoea trial on apnoea duration, HR and MAP, and Tukey's *post hoc* tests were used to locate specific differences. With regard to the remaining haemodynamic and lactate data, Student's paired *t*‐tests were used to compare baseline and apnoeic values.

Heritability estimates for the physiological variables were derived using only values obtained during the longest apnoea. As we have previously described (Missitzi et al., 2018), data obtained were analysed using the single‐factor ANOVA for each parameter to determine the significance of the differences between the mean MZ and DZ intrapair variance, taking into account twin type and pair factor. The geneticvariance(*F*) was calculated as *F* = MwDZ/MwMZ, where MwDZ and MwMZ stand for within‐pair mean squares for DZ and MZ, respectively, and derived from single factor ANOVAs. The associated degrees of freedom (d.f.) were determined by the number of MZ and DZ pairs. The following Clark equation based on intrapair variance was used to estimate heritability: *h* ^2^ = (*s* ^2^ DZ − *s* ^2^ MZ/*s* ^2^ DZ) × 100, where *s* ^2^ DZ is the variance of intrapair differences in DZ twins, and *s* ^2^ MZ is the variance of intrapair differences in MZ twins (Clark, 1956). The heritability estimate (*h* ^2^) was computed only if both of the following criteria were met: (1) (*F*) was found to be significant; and (2) the difference in the means (*t*′) and total variances (*F*′) between types of twins was found to be non‐significant (Christian, 1979). Statistical analyses were performed using Statistica v.10 (Statsoft) and Excel 2022 (Microsoft). The threshold for statistical significance was set a priori at α = 0.05.

## 3 RESULTS

### 3.1 Characteristics of the participants

General characteristics (mean ± SD) of MZ and DZ twins are illustrated in Table 1. Both groups of twins were similar regarding age, body mass, height, body mass index and FVC (*P* ≥ 0.529). Physical activity (based on work, sport and leisure‐time indices) was similar between the two groups of twins (*P* ≥ 0.096). Intrapair differences were also similar between zygosity groups (regarding work, sport and leisure‐time indices, *P* ≥ 0.051).

**TABLE 1:** General characteristics of monozygotic and dizigotic twins.

Parameter	MZ (*n* = 24)	DZ (*n* = 20)	*P*‐value
Age, years	24.7 ± 5.0	24.7 ± 4.4	0.972
Body mass, kg	77.0 ± 9.2	79.9 ± 8.6	0.741
Height, cm	176.6 ± 3.5	177.9 ± 7.0	0.529
BMI, kg/m^2^	24.7 ± 2.8	24.7 ± 2.5	0.957
FVC, L	5.8 ± 0.8	5.9 ± 0.9	0.901

*Note*: Values are mean ± SD. Abbreviations: BMI, body mass index; DZ, dizygotic; FVC, forced vital capacity; MZ, monozygotic.

### 3.2 Maximal apnoea duration

When MZ and DZ twins were analysed together, apnoea duration increased by 72% from the first (74.7 ± 35.2 s) to the fifth (123.5 ± 58.4 s) apnoeic trial (*P* < 0.0001). However, not everyone demonstrated the longest apnoea duration in the fifth apnoeic trial. Specifically, among the 44 participants, 32 (72%) exhibited the longest apnoea duration in the fifth trial, whereas the rest exhibited the longest apnoea duration in earlier apnoea trials (*n* = 6 in trial 4, *n* = 5 in trial 3, and *n* = 1 in trial 1).

### 3.3 Trial‐by‐trial measurements of apnoea duration, HR and MAP

Pooled data from all MZ and DZ twins showed a significant effect of trial for apnoea duration, HR and MAP (*P* < 0.001; Table 2). Apnoea duration progressively increased from trial 1 to trial 3 (all *P* < 0.001), with no difference between trials 3 and 4 (*P* = 0.584), followed by a further increase in trial 5 compared with trials 3 and 4 (*P* ≤ 0.00580). HR was reduced during all apnoeic trials compared with baseline (all *P* < 0.001 except trial 5: *P* = 0.0149). HR during the first apnoea did not differ from the following trials, whereas HR during the second apnoea was significantly lower than trials 3, 4 and 5 (*P* ≤ 0.00116). MAP increased during all apnoeic trials compared with baseline (all *P* < 0.001). MAP increased from trial 1 to trial 2 and remained elevated thereafter, except for a higher MAP in trial 5 compared with trial 2 (*P *= 0.00807).

**TABLE 2:** Apnoea duration, heart rate and mean arterial pressure across trials.

Parameter	Baseline	Trial 1	Trial 2	Trial 3	Trial 4	Trial 5	*P*‐value
Duration, s	–	74.7 ± 35.2	93.1 ± 45.7	109.1 ± 56.3	113.0 ± 56.5	123.5 ± 58.4	<0.001
*n*	–	44	44	44	44	44	
HR, beats/min	76.0 ± 11.0	61.4 ± 12.6	58.1 ± 10.5	62.8 ± 12.5	66.0 ± 13.4	66.9 ± 13.5	<0.001
*n*	44	42	42	43	43	44	
MAP, mmHg	97.2 ± 10.6	129.3 ± 18.3	133.9 ± 18.8	138.4 ± 19.7	139.3 ± 20.8	143.2 ± 19.6	<0.001
*n*	43	42	41	43	44	44	

*Note*: Values are the mean ± SD. Sample sizes varied across trials for HR and MAP because of missing data. HR and MAP values for trials represent end‐apnoeic measurements. The *P*‐values correspond to the main effect of trial. Abbreviations: HR, heart rate; MAP, mean arterial pressure.

### 3.4 Haemodynamic changes from baseline to the longest apnoea, oxygen saturation and blood lactate concentration

**FIGURE 2:**
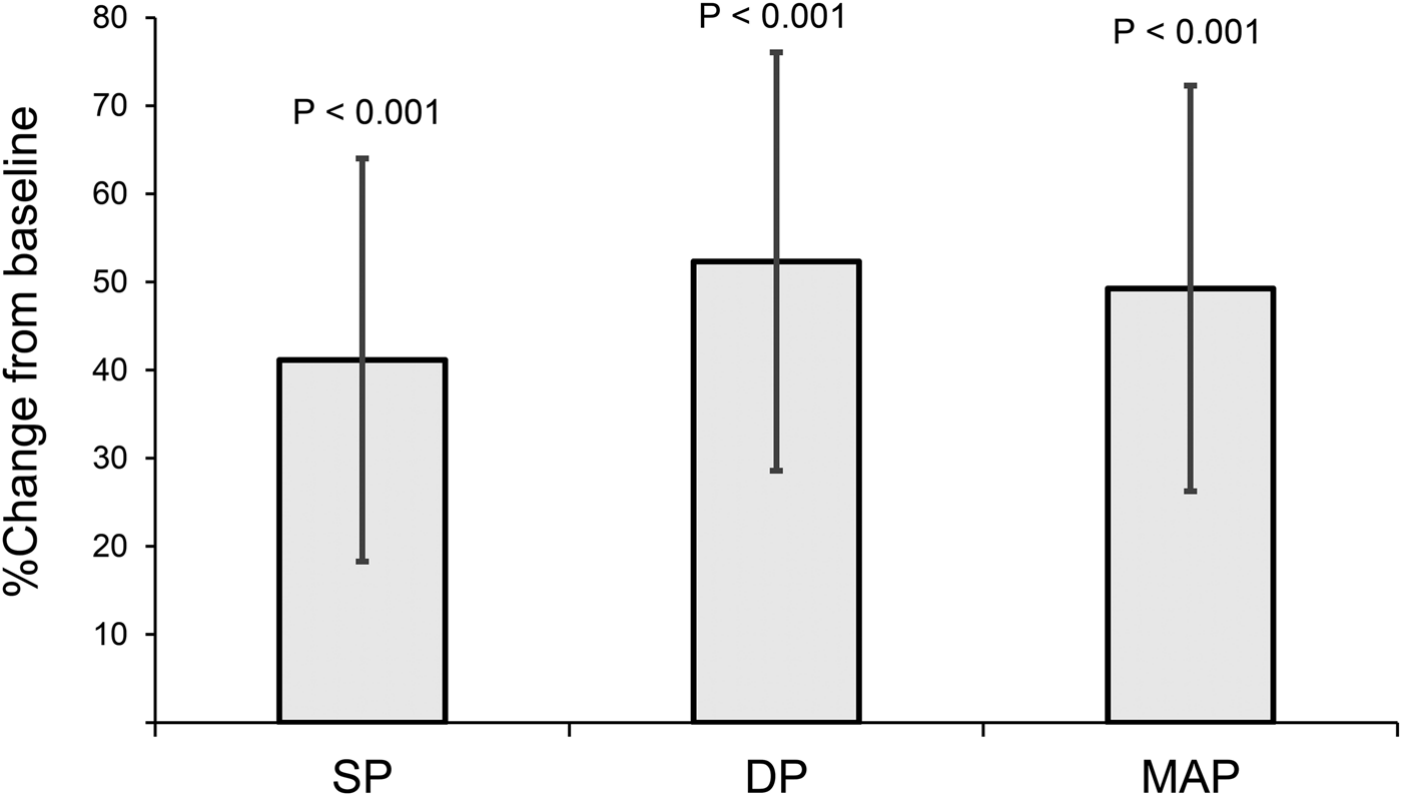
Mean percentage change from baseline to end‐apnoeic SP, end‐apnoeic DP and end‐apnoeic MAP. Bars represent mean values and error bars indicate ±SD. *n* = 43. The *P*‐values represent the statistical significance of differences between baseline and end‐apnoeic measurements obtained during the longest apnoea. Abbreviations: DP, diastolic pressure; MAP, mean arterial pressure; SP, systolic pressure.

**FIGURE 3:**
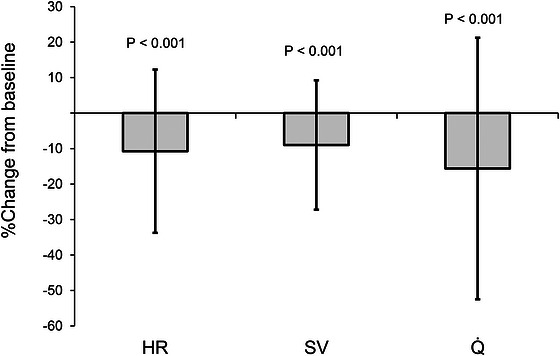
Mean percentage change from baseline to end‐apnoeic HR, end‐apnoeic SV and end‐apnoeic *Q̇*. Bars represent mean values and error bars indicate ±SD. For HR, SV and *Q̇*, *n* = 43, 42 and 42, respectively. The *P*‐values represent the statistical significance of differences between baseline and end‐apnoeic measurements obtained during the longest apnoea. Abbreviations: HR, heart rate; *Q̇*, cardiac output; SV, stroke volume.

Overall, SP increased from baseline (131 ± 12 mmHg) to the end of the longest apnea (184 ± 32 mmHg; 41%; *P <* 0.001). Similarly, DP and MAP increased from 76 ± 9 to 114 ± 16 mmHg (52%) and from 97 ± 10 to 144 ± 21 mmHg (49%), respectively (all *P* < 0.001; Figure 2). HR decreased by 11% from 76 ± 11 beats/min to 66.8 ± 13.8 beats/min (*P <* 0.001; Figure 3). SV decreased from 97.5 ± 16 to 87 ± 17 mL (11%) and *Q̇* from 7.4 ± 1.5 to 5.9 ± 1.6 L/min (20%) (all *P* < 0.001; Figure 3). TPR increased from 0.76 ± 0.18 to 1.40 ± 0.38 mmHg s/mL (82%) (*P* < 0.001).

No decrease in $S_{pO_{2}}$ (<96%) was observed in ∼50% of the participants owing to their short apnoea durations. The $S_{pO_{2}}$ decreased to 87.0% ± 9.8% in participants who exhibited desaturation during their longest apnoea. Because of this clustering at the upper end of the range, which resulted in a limited phenotypic variance, estimation of heritability for this variable was not feasible. A small but significant (*P* < 0.001) increase was observed in blood [La] 3 min after the fifth apnoeic bout (1.6 ± 0.6 mmol/L) compared with baseline (1.3 ± 0.4 mmol/L).

### 3.5 Heritability estimates

**FIGURE 4:**
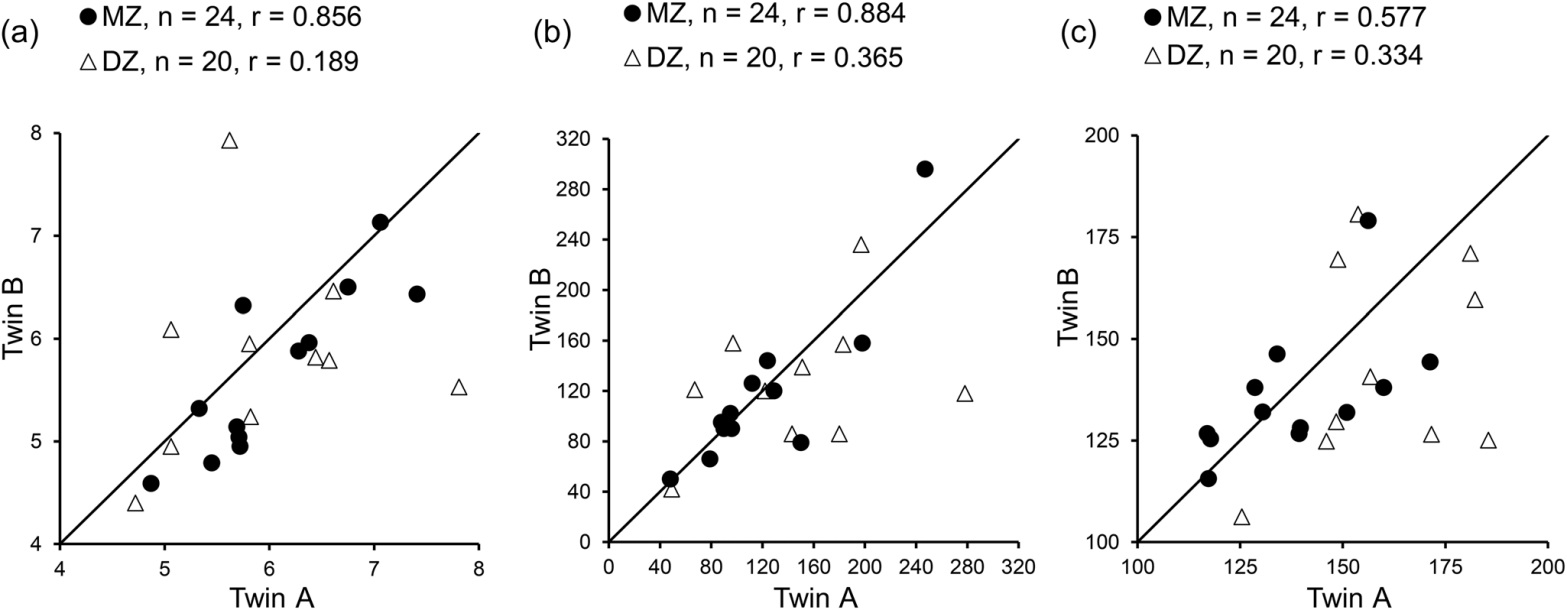
Individual values of deviation of forced vital capacity (in litres; a), maximal static apnoea duration (in seconds; b) and end‐apnoeic mean arterial pressure (in millimetres of mercury; c), in monozygotic (MZ) and dizygotic (DZ) twin pairs. *r* = intrapair correlation. *n* = 12 MZ pairs (filled circles) and *n* = 10 DZ pairs (open triangles).

Regarding maximal apnoea duration, FVC, end‐apnoeic MAP and end‐apnoeic DP, a significant intrapair correlation was found for MZ twins (*r* = 0.88, 0.85, 0.57 and 0.64; *P* < 0.001, < 0.001, 0.049 and 0.022, respectively) but not for DZ twins (*P* = 0.299, 0.600, 0.345 and 0.565, respectively) (Table 3). As illustrated in Figure 4, the values of the MZ twins for these parameters fell closer to the line of identity, whereas the values of the DZ twins were more scattered. Following the statistical analysis of the twin data, no differences were found between the means and the total variances of the two twin types (*t*′ and *F*′, respectively; Table 3). However, the genetic variance between the twin types was significant (*F* = 5.59, 4.42, 3.82 and 3.68; *P* = 0.00524, 0.0124, 0.0209 and 0.0236, respectively; Table 3). Hence, the heritability estimate (*h* ^2^) was computed and revealed that genetic factors explained 82%, 77%, 74% and 73% of the total variance in maximal static apnoea duration, FVC, end‐apnoeic MAP and end‐apnoeic DP, respectively. The heritability estimates for end‐apnoeic HR, SV, *Q̇*, SP and TPR were not computed as the statistical assumptions for these variables were not met (Table 3).

**TABLE 3:** Testing statistical hypotheses for the derivation of heritability estimates.

Trait	Intrapair correlation		Hypotheses			Heritability estimate (*h* ^2^)	Failed assumption
	MZ (*n* = 24)	DZ (*n* = 20)	*t*′ (*P*‐value)	*F*′ (*P*‐value)	*F* (*P*‐value)		
Maximal apnoea duration	0.88	0.36	0.73 (0.472)	1.03 (0.470)	5.59 (0.00524)	0.82	None
FVC	0.85	0.18	0.10 (0.921)	1.33 (0.285)	4.42 (0.0124)	0.77	None
End‐apnoeic MAP	0.57	0.33	1.99 (0.0633)	1.94 (0.0923)	3.82 (0.0209)	0.74	None
End‐apnoeic DP	0.64	0.56	1.14 (0.272)	2.17 (0.0720)	3.68 (0.0236)	0.73	None
End‐apnoeic HR	0.60	0.16	2.26 (0.0368)	2.20 (0.0532)	5.43 (0.00586)	–	Mean equality
End‐apnoeic SV	0.59	0.23	0.87 (0.398)	2.64 (0.0250)	5.81 (0.00454)	–	Total variance equality
End‐apnoeic *Q̇*	0.53	0.23	2.43 (0.0289)	3.45 (0.00724)	5.59 (0.00523)	–	Mean and total variance equality
End‐apnoeic SP	0.24	0.16	2.14 (0.0483)	1.97 (0.0774)	2.50 (0.0779)	–	Mean equality and genetic variance
End‐apnoeic TPR	0.29	0.35	0.34 (0.739)	1.64 (0.166)	1.48 (0.270)	–	Genetic variance

Note: t´, F´, and F tests indicate differences in the mean, the total variance, and the genetic variance, respectively, between the twin groups. Abbreviations: DP, diastolic pressure; DZ, dizygotic; FVC, forced vital capacity; HR, heart rate; MAP, mean arterial pressure; MZ, monozygotic; *Q̇*, cardiac output; SP, systolic pressure; SV, stroke volume; TPR, total peripheral resistance.

We re‐estimated *h* ^2^ for apnoea duration and MAP including only the non‐smoker participants and observed a very small genetic variance, resulting in a non‐significant *h* ^2^. However, this does not necessarily imply that smoking inflated within‐pair phenotypic variance in the DZ group. This re‐estimation was performed on a reduced sample (10 MZ pairs and 5 DZ pairs) and is therefore likely to be underpowered. Individual data are provided in the Supporting Information.

## 4 DISCUSSION

By applying the twin method, we demonstrated large heritability indices for maximal static apnoea duration (0.82), end‐apnoeic MAP (0.74), end‐apnoeic DP (0.73) and FVC (0.77). It should be noted that these heritability estimates reflect the proportions of the observed individual phenotypic differences attributable to genetic differences and do not imply biological determinism.

The present study appears to be the first to examine the extent to which individual differences in maximal static apnoea duration can be explained by genetic differences. Our results show that a substantial fraction (82%) of the interindividual variation in maximal static apnoea duration can be explained by genetic factors. Although the present study did not assess the mechanisms determining apnoea duration, of interest in this regard is the finding of previous twin studies demonstrating a genetic contribution to the hypoxic ventilatory response (Collins et al., 1978; Kawakami et al., 1982; Weil, 2003). Specifically, three factors have been proposed as the main determinants of maximal apnoea duration: (1) total body gas (O_2_ and CO_2_) storage capacity (lung and blood volumes, spleen size, haemoglobin mass and tissue gas stores); (2) oxygen‐conserving mechanisms (diving response and oxidative metabolic rate); and (3) mental drive (psychological tolerance) to withstand the increased urge to breathe (Bain et al., 2018; Hoiland et al., 2017; Klocke & Rahn, 1959; Linholm et al., 1999; Schagatay, 2009; Schagatay et al., 2001; Schneider, 1930). Owing to the underdeveloped psychological tolerance, the untrained apnoeist is typically unable to suppress the powerful respiratory urges arising from increased chemoreceptor activity (Elia et al., 2024), absence of inhibitory pulmonary afferent nerve activity (Flume et al., 1996), ongoing drive from the respiratory rhythm generator (Cooper et al., 2003; Parkes, 2006) and mounting involuntary breathing movements (Dejours, 1965). Regarding the chemoreflex component of the collective respiratory stress, studies in untrained apnoeists have shown that reducing pre‐apnoeic CO_2_ levels, either through hyperventilation or by dietary interventions, prolongs apnoea duration despite similar end‐apnoeic CO_2_ and lower O_2_ levels (Elia et al., 2022, 2024). These findings suggest that, in untrained individuals, the hypercapnic component of the chemoreflex drive might contribute more strongly to apnoea termination than hypoxaemia. Likewise, reducing the chemoreflex drive through pre‐apnoeic hyperoxic breathing in untrained apnoeists has been shown to result in a substantial increase in apnoea duration (Davidson et al., 1974; Engel et al., 1946; Klocke & Rahn, 1959), highlighting the significant contribution of hypoxic chemoreceptor stimulation to apnoea termination. Moreover, there is evidence to suggest that the hypoxic ventilatory response, an indirect measure of peripheral chemoreflex sensitivity, is a predictor of apnoea performance in the untrained apnoeist (Feiner et al., 1995). Recently, polymorphisms in *HMOX2*, which expresses the ‘O_2_ sensor’ heme oxygenase 2 in type I glomus cells, have been associated with divergent hypoxic ventilatory responses (Fabries et al., 2022), providing further support for a genetic predisposition in carotid body chemosensitivity. Given that maximal apnoea duration in the untrained apnoeist is significantly affected but not solely determined by the autonomic chemoreflex drive to breathe (Godfrey & Campbell, 1968), it could be assumed that the genetic influence on apnoea duration found in this study and the genetic influence on chemosensitivity found in previous twin studies (Collins et al., 1978; Kawakami et al., 1982; Weil, 2003) may be indirectly linked. Still, future studies are required to test the validity of this assumption and to directly evaluate the genetic underpinnings of the physiological and psychological determinants of apnoea performance through genome‐wide association studies, in both untrained and trained individuals.

Another new finding of the present study is the significant heritability index of MAP and DP during the final stages of maximal static apnoea. MAP is more strongly influenced by DP than by SP, particularly during diving bradycardia, where the cardiac cycle spends more time in diastole. Given that DP is considered an indirect marker of peripheral vasomotor tone (Goury et al., 2025; Smith & Madigan, 2018), these findings appear to be in line with those of Baranova et al. (2017), who demonstrated that subjects with bradykinin receptor B2 (*BDKRB2*) (CC), *ACE* (DD) and adrenoreceptor beta 2 (*ADBR2*) (GG, GA) genotypes showed the most intense peripheral vasoconstriction, hence, the most pronounced blood pressure changes during the diving response. These genes encode proteins of the renin–angiotensin and kinin–bradykinin systems, which play a significant role in the regulation of peripheral vasomotor activity and, consequently, TPR. Yet, contrary to the findings of Baranova et al. (2017), we found no genetic influence on end‐apnoeic TPR. This discrepancy might be explained by methodological differences between the studies, as TPR in the present study was calculated from Finometer‐derived pressure recordings and SV estimations obtained via the Modelflow method, whereas Baranova et al. (2017) used pulse transit time. Therefore, the non‐significant genetic component for calculated TPR should not necessarily be interpreted as evidence against a genetic component on vascular tone, given the significant heritability estimates observed for DP and MAP.

Variability was also observed regarding the trial in which participants performed their longest apnoea. Factors related to apnoea duration, such as psychological tolerance, chemoreflex sensitivity and respiratory drive, evolve across the apnoeic series (Mulder et al., 2025; Schagatay et al., 2001), and these changes might introduce systematic, non‐genetic variation that might inflate or mask genetic signals. To examine whether intra‐individual adaptation interacted with the twin design, heritability indices for apnoea duration, HR and MAP were also estimated at the first and fifth apnoeic trials, in addition to those estimated for the longest apnoea. Significant heritability estimates for apnoea duration were observed at both the first (*h* ^2 ^= 0.86) and the fifth apnoeic trial (*h* ^2^ = 0.80), suggesting that the observed genetic contribution to maximal apnoea duration was relatively consistent across trials and unlikely to represent an artefact of trial selection or adaptation. However, no significant heritability estimates were observed for end‐apnoeic HR in the first, fifth or longest apnoeic trials, indicating that the absence of a genetic influence on HR was unlikely to be explained entirely by trial‐by‐trial adaptation. Interestingly, a significant heritability estimate for end‐apnoeic MAP was observed only during the longest apnoeic trial and not during the standardised first of fifth trials, suggesting that a more extreme physiological stimulus might be required to reveal a genetic contribution to the arterial pressure response.

The statistical assumptions for derivation of the heritability estimates for end‐apnoeic HR, end‐apnoeic SV and end‐apnoeic *Q̇* were not met, indicating no genetic influence on these traits. Previous studies have focused on determining possible genetic contributions to cardiac performance using exercise as the environmental stressor. In contrast to our findings, there is evidence that a strong genetic component exists in the responses of SV, *Q̇* and HR to exercise (Bouchard et al., 2011; van de Vegte et al., 2019). However, the mechanisms governing the cardiovascular adjustments during exercise differ substantially from those during maximal static apnoea. Regardless of the environmental stimulus, MAP is the only cardiovascular variable with a sensor (i.e., baroreceptors), whereas *Q̇* (HR and SV) and TPR are considered the two main systemic components (‘effector organs’), the product of which defines arterial blood pressure. Future twin studies are needed to examine whether a genetic component influences the regulated haemodynamic variable (i.e., MAP) and the interplay among its regulator components (i.e., HR, SV and TPR) in response to divergent environmental stimuli.

Large lung volume is considered an important trait for apnoeic performance as more O_2_ can be stored and more CO_2_ can be diffused, ultimately leading to a slower arterial O_2_ desaturation and a delayed build‐up of CO_2_ in the blood (Schagatay, 2009). Moreover, it is generally accepted that elite apnoeists have larger lung volumes than untrained apnoeists (Bain et al., 2018; Elia & Gennser et al., 2021; Ferretti & Costa, 2003; Schagatay, 2009, 2014; Schagatay et al., 2012). Although there is evidence to suggest that apnoea training might lead to an increase in vital capacity (Schagatay, 2014), the significant heritability estimate for FVC found in this study provides support to a genetic contribution (Bain et al., 2018; Ferretti et al., 1991; Schagatay, 2009). Of note, the heritability estimate for FVC computed in this study was 77%, which is higher than heritability estimates generally reported in previous family aggregation and twin studies (ranging from 37% to 55%; Hukkinen et al., 2011). It is also worth mentioning that no correlation was found between maximal apnoeic time and FVC in this study. Notwithstanding, vital capacity and FVC have been correlated with apnoeic time in elite apnoeists (Bain et al., 2017; Koskolou et al., 2023; Schagatay et al., 2012), suggesting that O_2_ storage mechanisms play a pivotal role in apnoea performance in trained breath‐hold divers. In the present study, however, participants were untrained, and psychological tolerance to discomfort was likely to be the major determinant of apnoea duration. An intriguing question that arises is whether the wide scatter in the enhancement of apnoea performance observed among individuals who follow the same apnoea training programme might be explained, in part, by differences in their inherited lung volumes.

We observed a modest, albeit significant, increase in blood [La] from baseline following the apneic protocol in the present study. Similar increases in blood [La] (0.3 mmol/L) have been observed in elite apnoeists after maximal static apnoea lasting >5 min (Bain et al., 2016). Larger increases in blood [La] would, however, be expected during dynamic apnoeas (RodrÍguez‐Zamora et al., 2018) or when static apnoeas are performed with whole‐body immersion (Elia & Barlow et al., 2021).

## 5 LIMITATIONS

The present twin study is not without limitations, and these should be acknowledged when interpreting our findings. One limitation is the inclusion of nine smokers, most of whom (*n* = 6) were in the DZ twin group. Chronic smoking is associated with increased blood volume (Sotiridis et al., 2026), elevated baseline sympathoexcitation, increased vascular tone and, potentially, higher arterial pressure (Hering et al., 2010), which might have contributed to greater within‐group variance in the DZ group and inflated *h* ^2^ estimates. However, mechanisms governing the MAP response to apnoea appear largely redundant. For example, studies using β‐blockade to reduce cardiac output demonstrate maintenance of MAP via baroreflex‐mediated increases in TPR (Hoiland et al., 2017). Notably, chronic smoking has also been linked to blunted baroreflex function at rest (Middlekauff et al., 2013), although its effect during apnoea remains unclear. Despite these considerations, we did not observe a genetic component for baseline MAP, but we did find a significant *h* ^2^ for end‐apnoeic MAP during the longest apnoea, suggesting that stress‐induced MAP responses might be heritable, in part. Another limitation is the relatively small number of twin pairs (12 MZ and 10 DZ pairs), which limits statistical power for genetic modelling. Non‐significant heritability estimates for many variables might thus reflect type II error rather than a true absence of genetic influence, particularly when phenotype variance is reduced (e.g., minimal desaturation in many participants, which limits variability) or when the assumptions are not met (e.g., differences in means or total variances between twin types). All participants were male, which limits the generalisability of our findings to females. The findings of the present study should thus be interpreted with caution. Despite these limitations, the twin‐study design remains a valuable tool for examining potential genetic contributions to complex physiological traits.

## 6 CONCLUSION

On the grounds of the results obtained, it appears that a large portion of the variability observed in maximal static apnoea duration, end‐apnoeic arterial pressure and FVC can be explained by genetic differences. The significant heritability observed for apnoea duration likely reflects contributions from multiple genetically influenced physiological systems, including chemosensitivity and lung volume, but might also be influenced by unmeasured shared environmental factors, such as lifestyle, sleep or dietary habits. The importance of these findings can be viewed from a performance and health perspective, as they might explain a large part of the variability observed, respectively, in breath‐holding athletes and in patients suffering from obstructive sleep apnoea. Heritability estimates derived from twin studies provide a means of quantifying the relative contribution of genetic and environmental factors to physiological responses to extreme environmental stimuli. Further twin studies are warranted to examine possible genetic contributions to various protective mechanisms that tend to maintain body homeostasis when challenged by external environmental factors.

## AUTHOR CONTRIBUTIONS

Maria D. Koskolou conceived the research. Anastasios Makris, Maria D. Koskolou, Nickos Geladas and Vassilis Klissouras designed the research. Anastasios Makris and Alexandros Sotiridis performed the experiments and analysed the data. Kalliopi Gkouskou performed genetic analysis. Maria D. Koskolou and Anastasios Makris interpreted the results of experiments. Anastasios Makris drafted the manuscript, and Alexandros Sotiridis, Maria D. Koskolou, Nickos Geladas, Kalliopi Gkouskou and Vassilis Klissouras edited the manuscript. All authors approved the final version of the manuscript and agree to be accountable for all aspects of the work in ensuring that questions related to the accuracy or integrity of any part of the work are appropriately investigated and resolved. All persons designated as authors qualify for authorship, and all those who qualify for authorship are listed.

## CONFLICT OF INTEREST

None declared.

## FUNDING INFORMATION

None.

## Supporting information




Supporting Information

## Data Availability

The entire anonymised dataset has been made freely available as Supporting Information.
